# GM-CSF Treated F4/80^+^ BMCs Improve Murine Hind Limb Ischemia Similar to M-CSF Differentiated Macrophages

**DOI:** 10.1371/journal.pone.0106987

**Published:** 2014-09-09

**Authors:** Go Kuwahara, Hitomi Nishinakamura, Daibo Kojima, Tadashi Tashiro, Shohta Kodama

**Affiliations:** 1 Department of Cardiovascular Surgery, Faculty of Medicine, Fukuoka University, Fukuoka, Japan; 2 Department of Regenerative Medicine and Transplantation, Faculty of Medicine, Fukuoka University, Fukuoka, Japan; Bristol Heart Institute, University of Bristol, United Kingdom

## Abstract

Novel cell therapy is required to treat critical limb ischemia (CLI) as many current approaches require repeated aspiration of bone marrow cells (BMCs). The use of cultured BMCs can reduce the total number of injections required and were shown to induce therapeutic angiogenesis in a murine model of hind limb ischemia. Blood flow recovery was significantly improved in mice treated with granulocyte-macrophage colony-stimulating factor (GM-CSF)-dependent BMCs that secreted inflammatory cytokines. Angiogenesis, lymphangiogenesis, and blood flow recovery ratio were significantly higher in the GM-CSF-cultured F4/80^+^ macrophage (GM-Mø)-treated group compared with controls. Furthermore, Foxp3^+^ cell numbers and tissue IL-10 concentrations were significantly increased compared with controls. There was no significant difference in blood flow recovery between GM-Mø and M-CSF-cultured F4/80^+^ macrophages (M-Mø). Thus, GM-Mø were associated with improved blood flow in hind limb ischemia similar to M-Mø. The selective methods of culturing and treating GM-Mø cells similar to M-Mø cells could be used clinically to help resolve the large number of cells required for BMC treatment of CLI. This study demonstrates a novel cell therapy for CLI that can be used in conjunction with conventional therapy including percutaneous intervention and surgical bypass.

## Introduction

Despite the advances in surgical and endovascular techniques, treatment options for many patients with critical limb ischemia (CLI) remain limited. Approximately 20–30% of patients with CLI are not considered candidates for vascular or endovascular procedures, which often results in amputation as a final option [1]. Therefore, novel therapies are required to treat peripheral arterial disease (PAD) [2], [3]. In 1997, the successful isolation of endothelial progenitor cells (EPCs) from peripheral blood transformed the field of stem cell biology [4]. EPCs have been the focus of intensive research and have been proposed to be promising candidates for the induction of therapeutic angiogenesis in PAD. EPC transplantation improves neovascularization of ischemic hind limbs owing to their capacity to integrate new blood vessels and/or to secrete proangiogenic factors [5], [6]. However, outcomes of clinical studies using bone marrow-derived cells (including EPCs) transplanted into ischemic limbs of patients showed only minor improvements [7], [8]. In addition to poor host responses, cell therapy itself may also have inherent limitations, such as low survival rate of transplanted cells, cell age, and insufficient cell numbers. It is important for PAD patients to receive fewer cell injections as this reduces the need for repeated aspiration of bone marrow cells (BMCs).

We previously reported that CD11b-positive cells induced therapeutic improvements in a murine model of hind limb ischemia compared with conventional therapy using BMCs [9]. CD11b-positive cells might be useful for the treatment of PAD patients, as they possess a number of potential therapeutic mechanisms including angiogenesis and lymphangiogenesis. Consequently, *in vitro* selection of CD11b-positive cells reduces the total number of transplanted cells required for treatment. However, further characteristic analysis of CD11b-positive cells is required for their clinical use. In this study, we focused on monocytes, especially macrophages, which have angiogenic and inflammatory properties.

Monocytes are pluripotent progenitor cells that differentiate into immune effectors such as macrophages (Møs) or dendritic cells (DCs). Granulocyte-macrophage colony-stimulating factor (GM-CSF) can generate a population of cells with Mø and DC properties that are often used as a model of immature DCs. Although Møs are classically considered proinflammatory effector cells, they also have counter-inflammatory roles under certain conditions by releasing anti-inflammatory mediators. Depending on the microenvironment, Møs can acquire distinct functional phenotypes, referred to as classically activated, proinflammatory Møs (M1Mø) and alternatively activated, anti-inflammatory Møs (M2Mø) [10]. M2Mø are associated with anti-inflammatory and homeostatic functions linked to wound healing and tissue repair [11], [12]. GM-CSF induces different phenotypic changes in M1Mø lineage populations [13], [14], although the nature of the differences is unclear. Using cells cultured with GM-CSF, we assessed whether these cells could induce therapeutic angiogenesis in a murine model of hind limb ischemia.

## Methods

### Animal model and procedures

Twelve-week-old male C57BL/6N mice were purchased from Charles River Laboratories Japan Inc. The care of mice and experimental procedures complied with the “Principles of Laboratory Animal Care” (Guide for the Care and Use of Laboratory Animals, National Institutes of Health publication 86–23, 1985). The experimental protocol was approved by the Animal Care and Use Committee of Fukuoka University. Mouse hind limb ischemia was induced as previously described [9]. Briefly, the left femoral artery and vein were gently excised from the proximal portion of the femoral artery to the distal portion of the saphenous artery. The remaining arterial branches, including the perforator arteries, were also excised. The contralateral hind limb served as an internal control.

### Measurement of hind limb blood flow

Blood flow was monitored using a laser Doppler perfusion imager (Moor Instruments Ltd, Devon, UK). Blood flow was expressed as a ratio of the ischemic and non-ischemic hind limbs.

### Cell culture and enrichment of F4/80^+^ cells from GM-CSF cultured BMCs and flow cytometric analysis

After 8-week-old male C57BL/6N mice were sacrificed, BMCs from femurs and tibia were collected and cultured in RPMI 1640 medium, supplemented with 10% heat-inactivated fetal bovine serum supplemented with 100 U/mL penicillin and 100 µg/mL streptomycin containing either 10 ng/mL GM-CSF (R&D Systems, Minneapolis, MN, USA) or 10 ng/mL M-CSF (R&D Systems, Minneapolis, MN, USA). On day 7, adherent cells were harvested. F4/80^+^ macrophages were stained with an FITC-conjugated rat anti-mouse F4/80 antibody (eBioscience, San Diego, CA, USA) and anti-FITC MicroBeads (Miltenyi Biotec, Gladbach, Germany), and were enriched using a Magnetic Cell Sorter (Miltenyi Biotec, Gladbach, Germany). Expression of the CD11b, CD11c and F4/80 on GM-CSF or M-CSF treated-BMCs was assessed after 7 days in culture using the following antibodies: CD11b-FITC (BD Biosciences, San Jose, CA, USA), CD11c-PE (eBioscience, San Diego, CA, USA) and F4/80-APC (Biolegend, San Diego, CA, USA). Rat IgG2a kappa-APC (eBioscience, San Diego, CA, USA) was used as isotype control. Then, the cells were analyzed by flow cytometry (BD FACSVerse, BD Biosciences, San Jose, CA, USA) using FACSuite software. Data were analyzed using FlowJo software (Tree Star).

### In vivo tissue implant angiogenesis assay

The mice were randomly divided into five groups (n = 5 per group). Treatment groups received phosphate-buffered saline (PBS) (controls), 1×10^5^ M-CSF cultured BMCs, 1×10^5^ GM-CSF cultured BMCs, 1×10^5^ M-Mø cells, 1×10^5^ GM-Mø cells, or 5 µg of recombinant mouse IL-10 (R&D systems, Minneapolis, MN, USA) in a total volume of 100 µL in PBS, injected percutaneously into the thigh muscle at three points with a 27-gauge needle.

### Immunofluorescent staining

Mice were sacrificed at 7 days after surgery and thigh adductor muscles of the legs were removed, fixed in 10% formaldehyde solution, and paraffin embedded. Muscle fiber numbers were measured in sections stained with hematoxylin and eosin, and the mean counts from five separate fields in four distinct areas from each specimen were calculated. Capillary endothelial cells were identified by immunohistochemical staining with von Willebrand factor (vWF) antibody (Dako, Glostrup, Denmark). Capillary density was expressed as the ratio of vWF positive cells to myofibers per high power field (hpf) (magnification, ×400). Furthermore, lymphangiogenesis was assessed using the lymphatic vessel endothelial hyaluronan receptor (LYVE)-1 antibody (Relia Tech GmbH, Wolfenbüttel, Germany).

### Immunohistochemistry

The sections were stained immunohistochemically with anti-Foxp3 antibody (eBioscience, San Diego, CA, USA), then detected by diaminobenzidine (DAB) staining.

### Real-time PCR

Real-time PCR analysis was performed using the following primers: mouse (m) IL-10 forward: GCCAGAGCCACATGCTCCTA, mIL-10 reverse: GATAAGGCTTGGCAACCCAAGTAA, mouse tumor necrosis factor (mTNF)-alphaforward: TATGGCCCAGACCCTCACA, mTNF-alpha reverse: GGAGTAGACAAGGTACAACCCATC, mIL-6 forward: CCACTTCACAAGTCGGAGGCTTA, mIL-6 reverse: GCAAGTGCATCATCGTTGTTCATAC, mIL1beta forward: TCCAGGATGAGGACATGAGCAC, mIL-1beta reverse: GAACGTCACACACCAGCAGGTTA, mIL-12a forward: CCGGTCCAGCATGTGTCAA, mIL-12a reverse: CAGGTTTCGGGACTGGCTAAGA, mIL-12b forward: ACTCACATCTGCTGCTCCACAAG, mIL-12b reverse: CACGTGAACCGTCCGGAGTA mRplp0 forward: GGCAGCATTTATAACCCTGAAGTG and mRplp0 reverse: TGTACCCATTGATGATGGAGTGTG. Real-time PCR expression profile of cytokines was performed in M-CSF- or GM-CSF-cultured BMCs at day 7 using SYBR Green (TaKaRa, Japan) and LightCycler 2.0 systems (Roche, Basel, Schweiz), and relative quantification analysis was performed with LightCycler Software Version 4.1.

### Enzyme-linked immunosorbent assay (ELISA)

Supernatants of homogenized thigh muscles were tested for the presence of IL-10 using a commercially available ELISA kit (R&D Systems, Minneapolis, MN, USA) following the manufacturer’s protocol.

### Statistical analysis

Statistical significance was determined using the Student’s *t*-test, one-way ANOVA and Dunnett post hoc test. *P* values of <0.05 were considered statistically significant.

## Results

### Effect of implanted GM-CSF cultured BMCs in ischemic limb muscle

**Figure 1:**
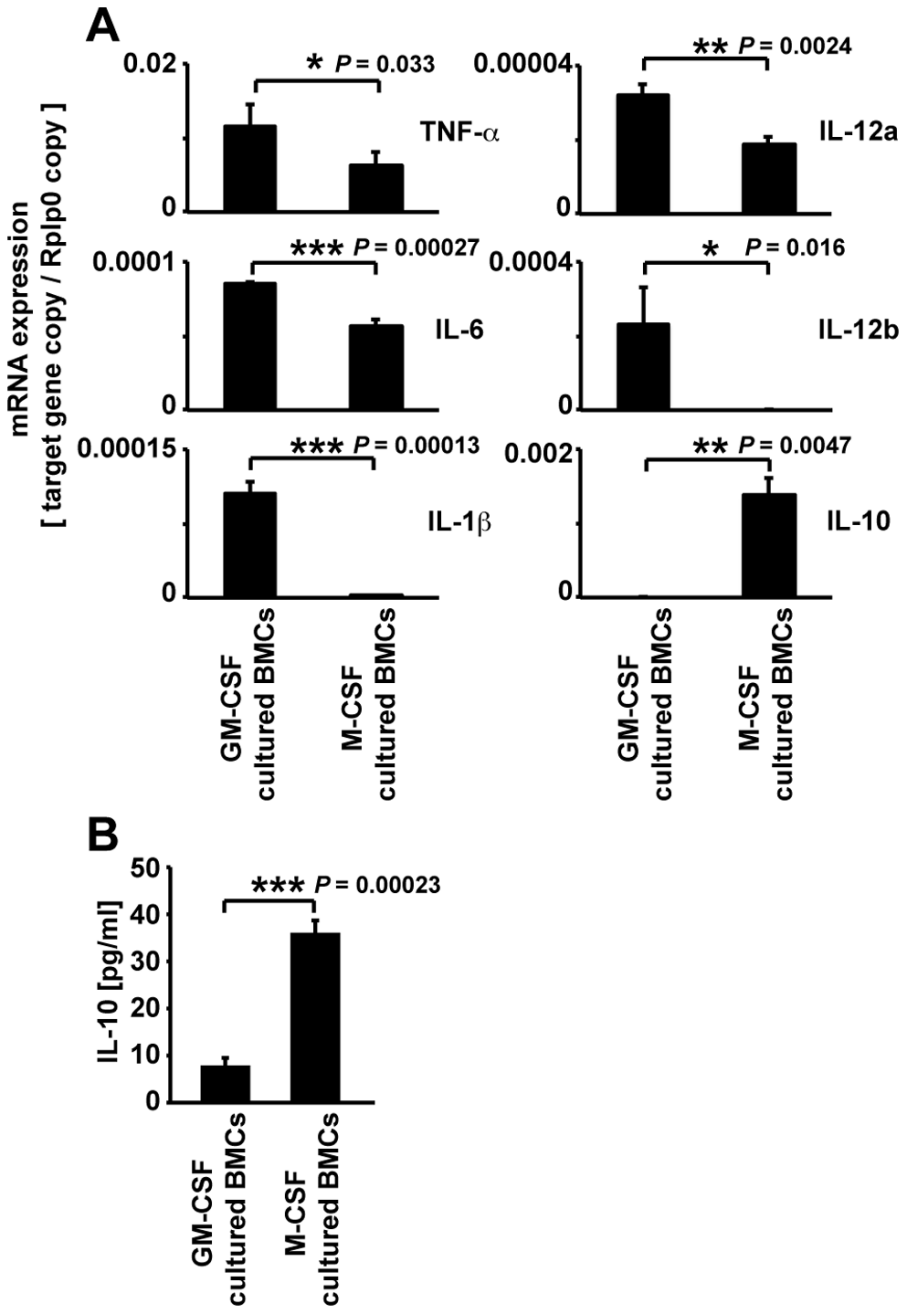
Expression of cytokine mRNA, and IL-10 protein levels in M-CSF- or GM-CSF-cultured BMCs. (A) The expression of proinflammatory cytokines (TNF-alpha, IL-6, IL-1beta, IL-12a, and IL12b) and anti-inflammatory cytokine (IL-10) mRNA in M-CSF- or GM-CSF-cultured BMCs. (B) IL-10 concentration in the supernatant of M-CSF- or GM-CSF-cultured BMCs. Data are expressed as mean ± SEM, ***P<0.001, **P<0.01, *P<0.05.

**Figure 2:**
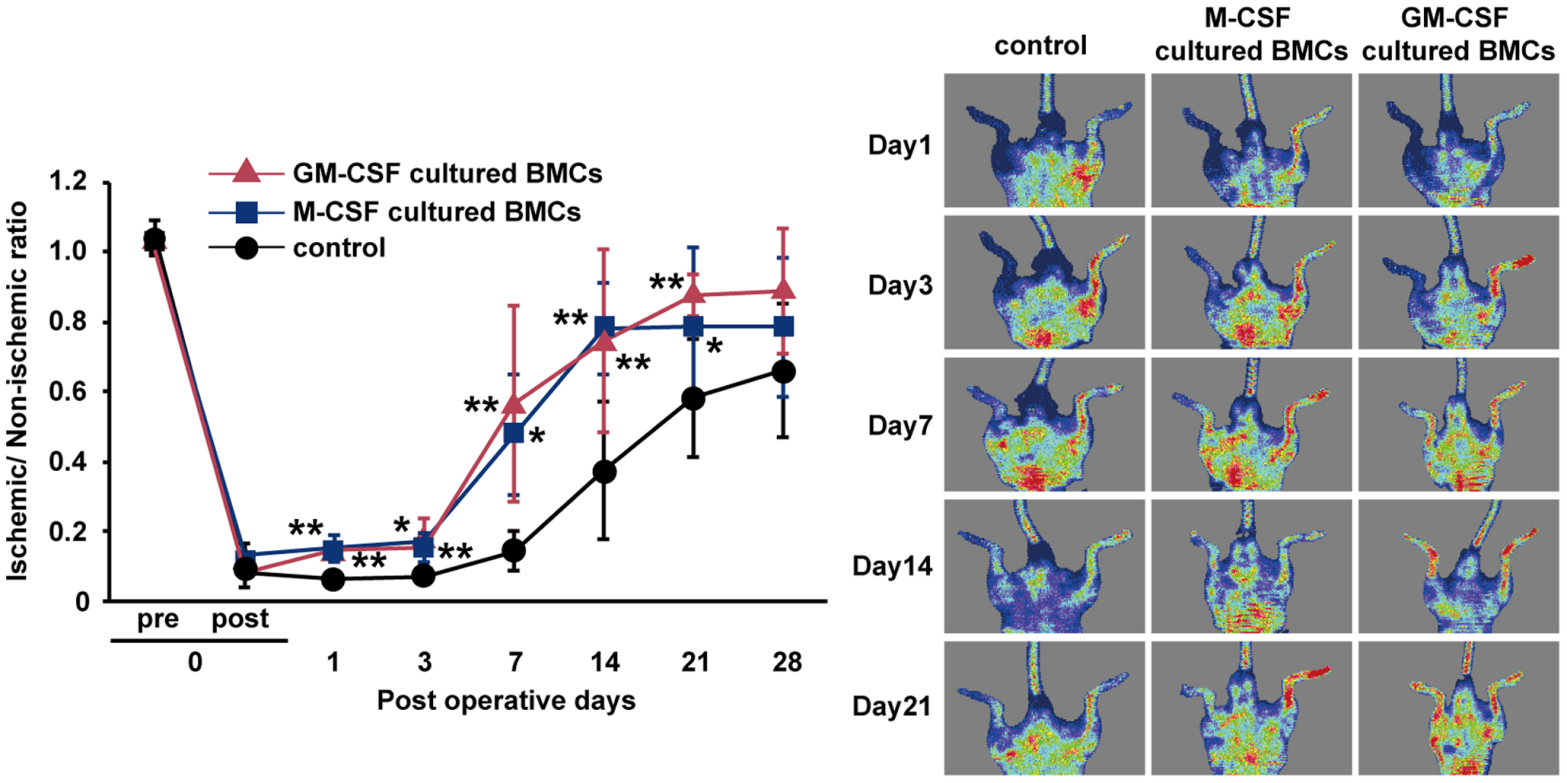
Recovery of blood flow in ischemic limbs treated with M-CSF-cultured BMCs and GM-CSF-cultured BMCs. Representative laser Doppler perfusion images taken at indicated intervals for the PBS control group, M-CSF-cultured BMCs group and GM-CSF-cultured BMC group. Data are expressed as mean ± SEM, n = 5–7/group, **P<0.01, *P<0.05.

**Figure 3:**
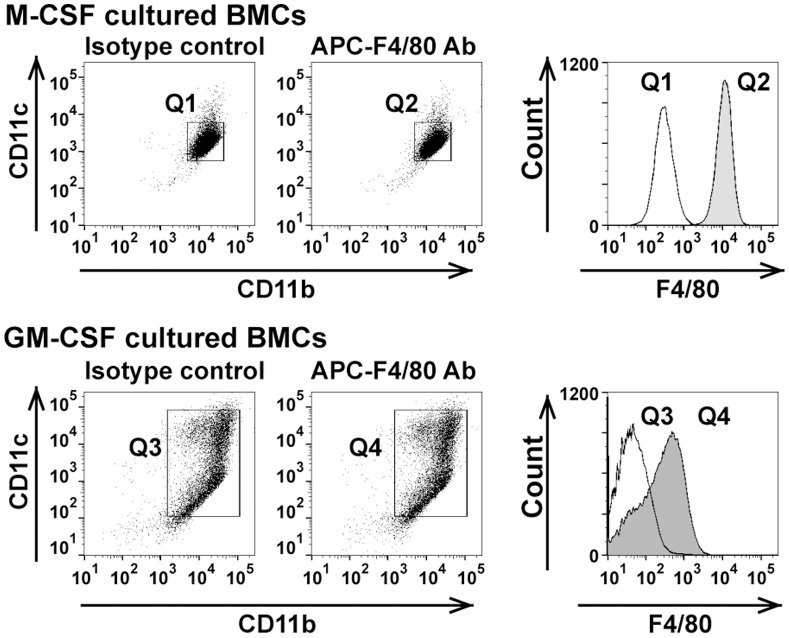
Flow cytometric analysis of F4/80^+^ macrophages from M-CSF- or GM-CSF- cultured BMCs. Data is representative of three independent experiments.

To examine the character of GM-CSF-cultured BMCs, we investigated the mRNA expression of TNF-alpha, IL-6, IL-1beta, IL-12a, IL-12b, and IL-10. As shown in Figure 1A, GM-CSF cultured BMCs expressed TNF-alpha, IL-6, IL-1beta, IL-12a, and IL-12b at significantly higher levels than M-CSF-cultured BMCs. However, IL-10 mRNA was higher in M-CSF-cultured BMCs compared with GM-CSF-cultured BMCs. The IL-10 protein level in M-CSF-cultured BMCs supernatant was 4-fold higher than in GM-CSF-cultured BMCs supernatant (Figure 1B). Because M-CSF-cultured BMCs produced IL-10, an anti-inflammatory cytokine, they were termed M2-like Møs. This pattern of cytokine expression indicated that GM-CSF-cultured BMCs were classically activated M1-like Møs and DCs. Using a mouse model of hind limb ischemia, blood flow recovery was significantly improved in mice treated with 1×10^5^ GM-CSF-dependent BMCs at 1, 3, 7, 14 and 21 days after the operative procedure similar to mice treated with 1×10^5^ M-CSF-dependent BMCs. At 28 days, the GM-CSF-dependent BMCs-treated group demonstrated a trend towards increased blood flow recovery compared with the control group, but this was not statistically significant (Figure 2). These effects were similar in the M-CSF-cultured BMCs-treated group. To investigate whether the Mø subtypes were involved in blood flow recovery in hind limb ischemia, we performed flow cytometric analysis of F4/80, a specific cell-surface marker for murine Mø. FACS analysis identified CD11b^+^CD11c^+^F4/80^+^ cells (Figure 3). Therefore, we isolated F4/80^+^ Møs (Figure 3; Q2 and Q4) by MACS sorting and used them to treat mice with hind limb ischemia.

### Recovery of blood flow in ischemic limbs treated with GM-CSF cultured F4/80^+^ Mø (GM-Mø)

**Figure 4:**
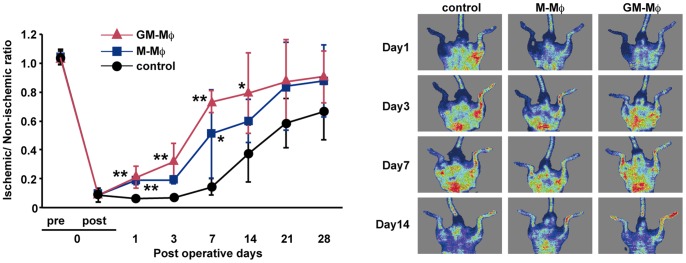
Recovery of blood flow in ischemic limbs treated with M-Mø and GM-Mø. Representative laser Doppler perfusion images were taken at indicated intervals for the PBS control group, M-Mø group, and GM-Mø group. Data are expressed as mean ± SEM, n = 5 group, **P<0.01, *P<0.05.

**Figure 5:**
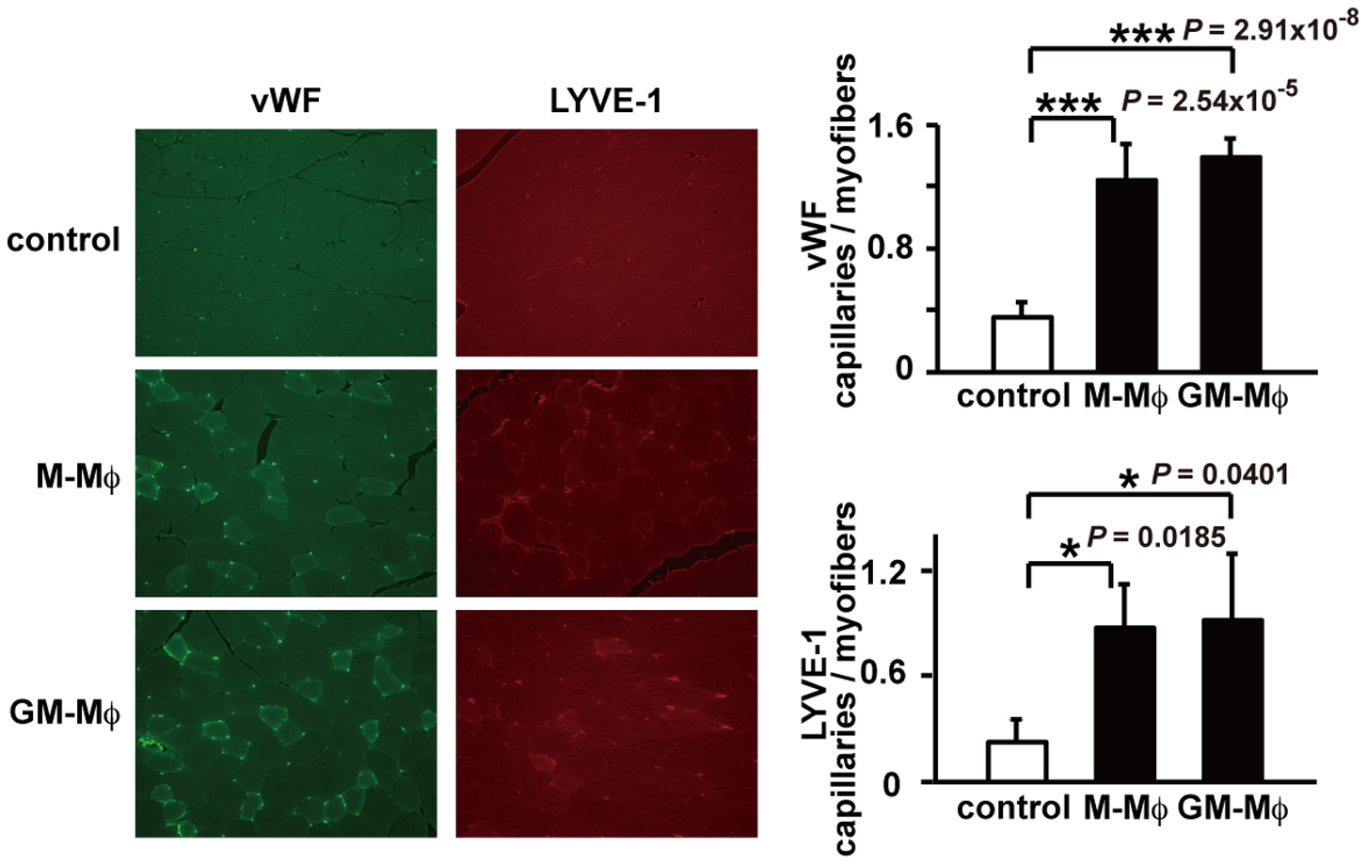
vWF and LYVE-1 staining in M-Mø and GM-Mø treated ischemic hind limbs. Representative photomicrographs (original magnification ×400). vWF positive and LYVE-1 positive cells are stained green and red, respectively (***P<0.001, **P<0.01, *P<0.05).

To determine whether enriched GM-CSF cultured F4/80^+^ Mø (GM-Mø) contributed to improved blood flow in the ischemic hind limbs, we measured the recovery of blood flow in ischemic hind limbs treated with purified F4/80^+^ Mø. Although 5×10^4^ or 1×10^4^ GM-Mø did not improve the blood flow in hindlimb ischemia compared with control significantly, 1×10^5^ GM-Mø improved as the lowest cell numbers (data not shown). Therefore, mice were treated with 1×10^5^ GM-Mø or PBS for controls. At the earliest operative time point (day 1), the blood flow recovery ratio was higher in the GM-Mø-treated group compared with the control group. Moreover, the blood flow recovery was significantly improved in the GM-Mø-treated group compared with controls at 1, 3 7, and 14 days after the operative procedure, similar to the M-Mø-treated group. At 21 and 28 days, there was no significant difference in blood flow recovery between the GM-Mø-treated group and control group (Figure 4). After induction of hind limb ischemia, the GM-Mø-treated group had significantly increased numbers of vWF-positive cells and LYVE-1-positive cells compared with the control group. The capillary density, representing angiogenesis, was significantly higher in the GM-Mø-treated group compared with the control group. Furthermore, the capillary density, representing lymphangiogenesis, was significantly upregulated in the GM-Mø-treated group compared with the control group. There was no significant difference in vWF-positive or LYVE-1-positive cell numbers between the M-Mø-treated group and GM-Mø-treated group (Figure 5).

### Effect of GM-Mø or IL-10 treatment in hind limb ischemia

**Figure 6:**
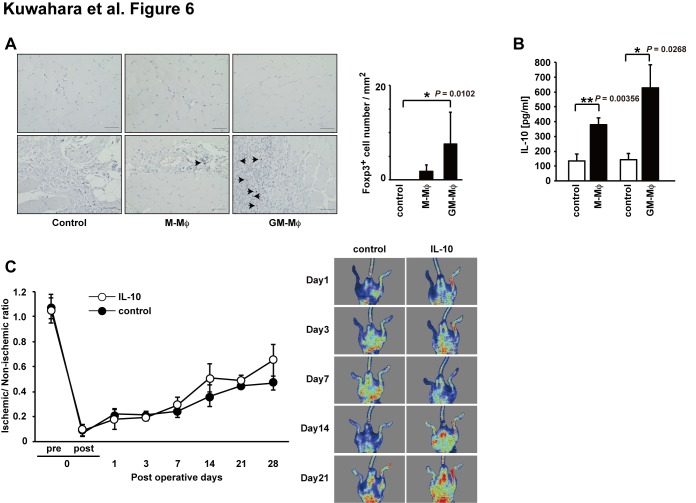
Foxp3 expression in M-Mø or GM-Mø treated ischemic hind limbs and the recovery of blood flow in ischemic limbs treated with IL-10. (A) Photomicrographs show representative DAB staining for Foxp3 (scale bar = 50 µm). The number of Foxp3 positive cells was counted in five random images for each mouse. The thigh muscle of each group was isolated 7 days after the induction of ischemia. (B) IL-10 levels in thigh muscle after limb ischemia at 7 days after intramuscular injection of indicated cells (data are expressed as mean ± SEM, n = 3 group, ***P<0.001, **P<0.01, *P<0.05). (C) Recovery of blood flow in ischemic limbs treated IL-10. Representative laser doppler perfusion images were taken at indicated intervals for the PBS control groups and IL-10 treated groups. Data are expressed as mean ± SEM, n = 5–7/group.

Recently, the contribution of regulatory T cells (CD4^+^CD25^+^Foxp3^+^ cells; Treg) in arteriogenic responses was demonstrated by major changes in Foxp3^+^ Treg numbers in ischemic muscle [15], [16]. To evaluate the degree of Treg infiltration, we performed Foxp3 staining in hind limb tissues at postoperative day 7. As shown in Figure 6A, Foxp3^+^ Treg cells in the GM-Mø-treated group tissues were increased significantly compared with control tissues. Furthermore, IL-10 was increased in the hind limb tissues of the GM-Mø-treated group compared with the control group (Figure 6B). IL-10 dose not induce the Foxp3 positive Treg directly, but it is key mediator of Treg function [17], [18]. Therefore, to identify whether the IL-10 directly induce the recovery of blood flow in ischemic hind limb, we performed simply administration of IL-10 in hind limb ischemia mouse model [19]. Blood flow recovery was not significantly improved in mice treated with IL-10 treatment at 1, 3, 7, 14 and 21 days after the operative procedure compared with the control group (Figure 6C). These results suggested that blood flow improvement in ischemic hind limbs by GM-Mø was mediated primarily through the activation of Treg and IL-10 secreted by Treg or other cells.

## Discussion

This study described for the first time the induction of blood flow recovery by GM-CSF-cultured BMCs treatment in a mouse model of hind limb ischemia. Cultured BMCs treatment reduced the total number of cell injections required and improved the early operative points of blood flow compared with conventional therapy using BMCs [9]. Previous studies suggested that EPC populations could be grown from mononuclear cells (MNCs) [6] or purified populations of CD34- or AC133-positive hematopoietic cells [20]. In addition, CD14-positive MNCs have been used as starting populations for the cultivation of EPCs [6]. The blood flow recovery of hind limb ischemia was induced by 5×10^5^ EPCs/mouse [20], but we demonstrated that 1×10^5^ GM-CSF-cultured BMCs/mouse could significantly increase blood flow recovery compared with controls. The need for lower numbers of cells is helpful in reducing the need for repeated aspiration of BMCs in clinical trials.

In mice, M-CSF-cultured BMCs are frequently used as a convenient population to study Mø functions and signaling [13]. In contrast, GM-CSF-treated BMCs give rise to cells used as a model for DCs, although they express Mø markers, and are excellent osteoclast precursors with a transcriptome closer to Møs than to DCs [21], [22]. In the current study, M-CSF-cultured BMCs expressed IL-10, thus these cells were characterized as M2 like Mø. In contrast, GM-CSF-cultured BMCs expressed IL-6, TNF-alpha IL-1beta, IL-12a and IL-12b indicating they were M1-like Mø and DCs. Although distinct Mø subpopulations are clearly observed at different phases of the immune response to infection and injury, the regulation of the phenotypic polarization of these remarkably plastic cells remains poorly characterized. To compare Mø subtypes regarding angiogenesis and lymphangiogenesis in ischemic hind limbs, we used a pan-Mø marker, F4/80, in GM-CSF-cultured BMCs.

Mø have a wide variety of biological functions, including participation in both pro-angiogenic and anti-angiogenic systems. This dual function of Mø is largely dependent on their polarization [23]. The anti-inflammatory cytokine IL-10 especially has a significant influence on Mø polarization and promotes neovascularization in a mouse model of oxygen-induced retinopathy [24]. M-Mø that produce other cytokines and chemokines including IL-10, and 1×10^5^ M-Mø cells improved the blood flow recovery of ischemic hind limbs. Furthermore, in tissues at day 7 after M-Mø treatment, the IL-10 concentration was significantly higher compared with controls resulting in the induction of angiogenesis and lymphangiogenesis. Although GM-Mø produced lower IL-10 levels than M-Mø cells, they expressed proinflammatory cytokines (TNF-alpha, IL-6, IL-1beta, IL-12a, and IL-12b), indicating they were M1 like Mø cells. Despite the M1 like Mø character of GM-Mø, when used to treat ischemic hind limbs they improved blood flow after the induction of ischemia similar to M-Mø. Moreover, the concentration of IL-10 and the number of Foxp3^+^ cells increased in the ischemic hind limbs compared with controls and GM-Mø-treated groups (Figure 6A, 6B). Although IL-10 dose not induce Treg directly, little information is available on the role of effector Th1, Th2, Th17, Tr1, and Treg subsets in the regulation of angiogenesis and lymphangiogenesis [18], [25], [26]. Furthermore, the presence of Foxp3^+^ Treg suggests that inflammatory cytokines and chemokines in hind limb ischemia produced by GM-Mø may attract or induce Treg. Recently, a study by Sharir et al., Treg plays an important role in blood flow recovery after inducible hind limb ischemia, and that IL-10 plays a mediator in these effects [27]. IL-10 was shown to be important in the improved blood flow [24], [28]. Their activation may then modulate the immunoinflammatory response to ischemic injury, leading to alteration of post-ischemic angiogenesis and lymphangiogenesis. Jetten et al. also demonstrated that local delivery of 5×10^5^ polarized M1 Mø cells (induced by lipopolysaccharide and interferon-gamma) or M2 (induced by IL-4 and IL-10) Mø subsets improved reperfusion recovery in a mouse hind limb ischemia model at day 14, although they did not examine the mechanism involved [29], [30]. Moreover, Troidl et al. demonstrated that a forced shift toward M1 and M2 (by intravenous injections of IL-10 or IL-4/IL-13) macrophages improved the arteriogenic response [31]. Thus, the selective methods of culturing and treating M1Mø cells similar to M2Mø cells could be used clinically to help resolve the large number of cells required for BMCs therapy of CLI. Both M1Mø and M2Mø have important roles in improved blood flow recovery after hind limb ischemia.

In humans, M-CSF-treated blood monocytes are often used to generate monocyte-derived Mø as a model for tissue Mø [32], [33]. GM-CSF-treated monocytes are widely used as a model system for DC development and function, although bioinformatic analysis of their transcriptome indicates that they are closer to Møs than to DCs [34]. Similarly, BM-derived M-CSF or GM-CSF-cultured M1Mø and M2Mø categorization and characterization have been defined, thus the results from the current study could help develop a novel cell therapy for treatment of CLI in combination with conventional therapy including percutaneous intervention and surgical bypass.
